# Pan-cancer analysis reveals correlation between RAB3B expression and tumor heterogeneity, immune microenvironment, and prognosis in multiple cancers

**DOI:** 10.1038/s41598-024-60581-x

**Published:** 2024-04-30

**Authors:** Xu-Sheng Liu, Ya-Lan Chen, Yu-Xuan Chen, Rui-Min Wu, Fan Tan, Ya-Lan Wang, Zi-Yue Liu, Yan Gao, Zhi-Jun Pei

**Affiliations:** 1 Department of Nuclear Medicine, Hubei Provincial Clinical Research Center for Precision Diagnosis and Treatment of Liver Cancer, Taihe Hospital, Hubei University of Medicine, Hubei, 442000 China; 2 Department of Gastroenterology, The Affiliated Hospital of Hebei University, Baoding, 071000 Hebei China; 3 Hubei Provincial Clinical Research Center for Umbilical Cord Blood Hematopoietic Stem Cells, Taihe Hospital, Hubei University of Medicine, Shiyan, 442000 Hubei China; 4 Hubei Key Laboratory of Embryonic Stem Cell Research, Shiyan, 442000 Hubei China

**Keywords:** RAB3B, Pan-cancer, Diagnosis, Prognosis, Immune infiltration, Cancer genomics, Cancer microenvironment, Tumour biomarkers, Tumour heterogeneity, Tumour immunology

## Abstract

RAB3B is essential for the transportation and secretion within cells. Its increased expression is linked to the development and progression of various malignancies. However, understanding of RAB3B’s involvement in carcinogenesis is mostly limited to specific cancer subtypes. Hence, exploring RAB3B's regulatory roles and molecular mechanisms through comprehensive cancer datasets might offer innovative approaches for managing clinical cancer. To examine the potential involvement of RAB3B in the development of cancer, we analyzed data from various sources including The Cancer Genome Atlas (TCGA), Genotype-Tissue Expression Project (GTEx), cBioPortal, HPA, UALCAN, and tissue microarray (TAM). Using bioinformatics techniques, we examined the correlation between RAB3B expression and prognosis, tumor heterogeneity, methylation modifications, and immune microenvironment across different cancer types. Our findings indicate that elevated RAB3B expression can independently predict prognosis in many tumors and has moderate accuracy for diagnosing most cancers. In most cancer types, we identified RAB3B mutations that showed a significant correlation with tumor mutational burden (TMB), mutant-allele tumor heterogeneity (MATH), and microsatellite instability (MSI). Abnormal DNA methylation patterns were also observed in most cancers compared to normal tissues. Additionally, we found significant correlations between RAB3B expression, immune cell infiltration, and immune scores across various cancers. Through pan-cancer analysis, we observed significant differences in RAB3B expression levels between tumors and normal tissues, making it a potential primary factor for cancer diagnosis and prognosis. The IHC results revealed that the expression of RAB3B in six types of tumors was consistent with the results of the pan-cancer analysis of the database. Furthermore, RAB3B showed potential associations with tumor heterogeneity and immunity. Thus, RAB3B can be utilized as an auxiliary diagnostic marker for early tumor detection and a prognostic biomarker for various tumor types.

## Introduction

With the extension of human lifespan and changes in lifestyle, cancer has become one of the major health challenges worldwide^1^. The occurrence of cancer is closely related to various complex factors, including genetic mutations, environmental exposure, and lifestyle habits. In recent years, remarkable progress has been made in cancer research^1–3^. However, there are still many uncertainties regarding the mechanisms of cancer development and treatment methods.

Pan-cancer analysis is a comprehensive method that integrates various cancer data. By analyzing the genomes of different types of cancer patients, it aims to explore the common characteristics and differences of cancer^2,4–7^. Among them, genetic mutations are one of the important driving factors for the occurrence and development of cancer^8,9^. As far as cancer diagnosis and treatment go, genomic heterogeneity plays a crucial role in determining the accuracy of cancer diagnosis^10,11^. DNA methylation of promoters, as an important epigenetic modification, has been shown to be closely associated with the occurrence and development of various cancers. Abnormal methylation patterns often accompany tumor development, leading to gene hypermethylation or hypomethylation, which can affect gene expression and function^12,13^. Combating cancer is significantly influenced by the immune system. In recent years, the detection of immune cell infiltration has become an important indicator for assessing the prognosis and treatment efficacy of cancer patients^14–18^. Certain types of tumors are linked to the development of immune checkpoints, which are molecular mechanisms responsible for regulating immune responses and can become dysregulated ^19,20^. ImmuneScore, as a critical indicator for measuring tumor immune response, has played a key role in predicting patient prognosis and developing personalized treatment strategies^21,22^. Therefore, by systematically studying the potential connections between RAB3B expression, immune cell infiltration, immune checkpoints, and immune scoring in pan-cancer, new strategies and targeted therapies can be provided for cancer treatment.

There are several subfamilies of the Ras superfamily of small molecule GTP binding proteins, but the RAB protein family represents the largest. Changes in Rab proteins and their effectors are associated with various human diseases, including neurodegeneration, infection, endocrine disorders, and cancer^23,24^. Ras-Related Protein Rab-3B (RAB3B) belongs to the Ras-related protein family and is mainly involved in intracellular transport and secretion processes. There has been an increase in research on RAB3B's relevance to cancer over the past few years. It has been suggested that high levels of RAB3B are associated with cancer development and occurrence, including gliomas , prostate cancer, breast cancer, pancreatic cancer, and cancer stem cells^25–29^. Additionally, studies have found that RAB3B can act as a hub gene in the diagnosis and prognosis prediction of lung adenocarcinoma, glioma, prostate cancer, gastric cancer, and colorectal cancer^30–35^. Research on RAB3B in cancer, however, is mostly confined to specific types. Developing new cancer therapies can therefore be improved by studying RAB3B's molecular mechanisms and regulatory functions in pan-cancer datasets.

**Figure 1:**
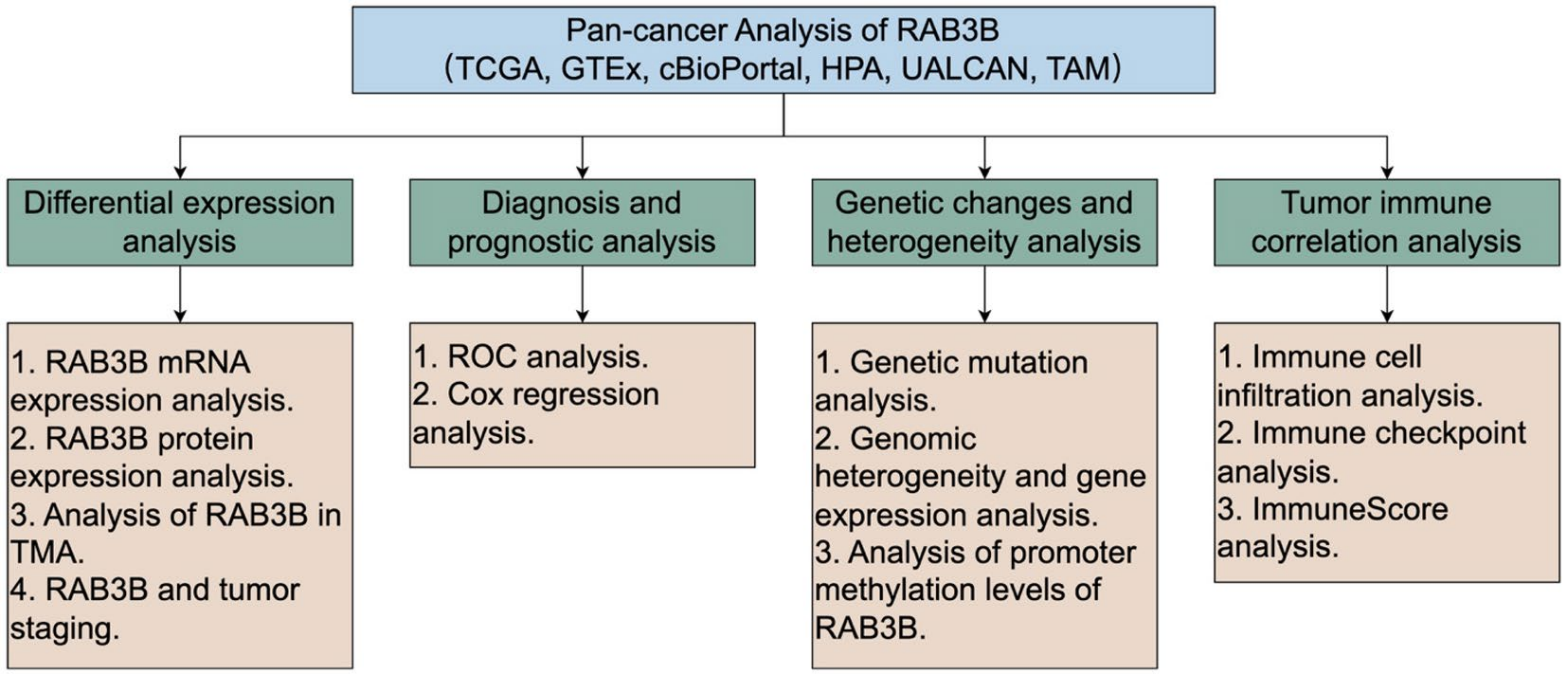
Experimental design. The figure was created by Figdraw (www.figdraw.com).

For this research, we employed data from databases like TCGA and GTEx to perform a comprehensive examination of RAB3B across various types of cancer. Our analysis focused on exploring the expression levels and prognostic significance of RAB3B in pan-cancer. In addition, we examined the possible correlation between genetic variations in the RAB3B gene and the formation of tumors. This involved studying the mutation status of the RAB3B gene across various types of cancer, as well as investigating the association between its expression and factors such as tumor mutation burden (TMB), mutant-allele tumor heterogeneity (MATH), microsatellite instability (MSI), and DNA methylation. In order to gain a better understanding of the significance of RAB3B in tumor immune therapy, we conducted an analysis to examine its expression in relation to immune cell infiltration, immune checkpoint-related genes, and immune scoring, thereby further investigating the correlation between RAB3B and tumor immunity. Through our analysis of various types of cancer, we have gained a thorough comprehension of how RAB3B contributes to their development, broadening the possibilities for utilizing RAB3B in the treatment of cancer. The research process is shown in Fig. 1.

## Method

### Analysis of RAB3B expression differences and ROC curve analysis in pan-cancer

We downloaded and organized RNA-seq data (in TPM format) for 33 types of cancer projects from the TCGA database (https://portal.gdc.cancer.gov)^36^ and processed TCGA and GTEx RNA-seq data (in TPM format)^37^ using the Toil pipeline^38^ from the UCSC XENA database (https://xenabrowser.net/datapages). From this database, we extracted TCGA data for pan-cancer analysis and corresponding normal tissue data from GTEx to analyze RAB3B expression in various tumors. The statistical R package (version 3.6.4), "pROC", was utilized to execute the Receiver Operating Characteristic (ROC) assessments on the gathered pan-cancer dataset from TCGA. The predictive accuracy of RAB3B gene expression in the pan-cancer setting was represented by the Area Under Curve (AUC) values deduced from ROC assessments, with a significant threshold set at an AUC value surpassing 0.7 to ascertain reliability.

### The analysis of the clinical proteomic tumor analysis consortium (CPTAC)

The CPTAC database (https://proteomics.cancer.gov/programs/cptac) comprises both genomic and proteomic data, allowing for examination and analysis of gene expression and expression patterns across different tumor types. Analysis of this data has the potential to reveal candidate biomarkers or therapeutic targets for various cancer types. Additionally, we examined the mRNA and protein levels of RAB3B by utilizing the Cancer Proteogenomic Data Analysis Site (cProSite) database (https://cprosite.ccr.cancer.gov)^39^.

### Protein expression scores from the human protein atlas

The HPA, also known as the Human Protein Atlas, is an extensive database that provides details about the localization of proteins in human tissues and cells (https://www.proteinatlas.org/)^40^. To further explore the differential expression of RAB3B in terms of protein, we employed the HPA database to showcase the level of staining for RAB3B in both tumor and normal tissues across 10 different organs, namely breast, colon, endometrium, liver, lung, ovary, pancreas, testis, thyroid, and bladder.

### Pan-cancer tissue microarray (TAM) and immunohistochemistry

Human pan-cancer TMAs (ZL-MTU122) were purchased from Shanghai Zhuoli Biotechnology Co., Ltd (Zhuoli Biotechnology Co., Shanghai, China). The TMA chip contained tumors from 20 cancers and matched adjacent normal/normal tissues^41^. Immunohistochemical (IHC) staining was performed according to the previously described method^42^. Then, differences in expression of RAB3B (1/50, 15774–1-AP, proteintech) in pan-cancer were assessed using digital image analyses (DIA) techniques from the fully automated VIS DIA VisioMorph system (Visiopharm®, Hoersholm, Denmark)^43^.

### Exploring the relationship between RAB3B gene expression and clinical features

In this study, we used R software to investigate the potential relationship between the expression of the RAB3B gene and various tumor stages in different cancers. Differences between two pairs were significantly analyzed using an unpaired student's t-test, and differences were tested across multiple groups of samples using analysis of variance.

### Pan-cancer prognostic assessment of RAB3B

To acquire reliable predictive gene expression data, the pan-cancer expression data and clinical information were filtered by removing incomplete survival information and status samples. Cox regression analysis was then conducted using a single variable and the “forestplot” R package was used to display P values, HR, and 95% CI via a graphical forest plot. The predictive significance of RAB3B in various types of cancer was assessed in four clinical measures: overall survival (OS), progression-free survival (PFS), disease-specific survival (DSS), and disease-free survival (DFS). A statistical significance of p < 0.05 was considered significant for the evaluation.

### Pan-cancer analysis of RAB3B gene changes

cBioPortal is a website (http//www.cBioPortal.org/) that offers researchers multidimensional visualization data by housing all the tumor gene information found in the TCGA database^44,45^. We analyzed data from 30 cancer types in cBioPortal, totaling 10, 953 samples. In OncoPrint and Cancer Type Summary, the analysis was conducted on the mutations of the RAB3B gene in all tumors, examining their types and frequencies. Heat maps in OncoPrint exhibit the alterations in target genes, including mutations, copy numbers, and expression, across all samples. Furthermore, the histogram in the 'Cancer Type Summary' showcases the frequency of mutations in target genes across various types of cancer.

### Genomic heterogeneity and gene expression analysis

TMB (Tumor Mutational Burden) refers to the number of mutations in the genome of tumor cells, usually measured in terms of mutations per megabase (mut / Mb). MATH (Mutant-Allele Tumor Heterogeneity) refers to the degree of heterogeneity of mutant alleles within tumor cells. Microsatellite instability (MSI) refers to the instability of microsatellites in tumor cells, which refers to the insertion or deletion of microsatellite sequences during DNA replication. The above three are indicators related to the heterogeneity of the tumor genome. Tumor genomic heterogeneity refers to the presence of different genomic variations in tumor cells. Mutation of tumor genes is an important driving factor for the occurrence and development of tumors. TMB, MATH, and MSI are closely related to tumor gene mutations, and tumors with high TMB, high MATH, and high MSI typically have more gene mutations. These mutations may affect the proliferation, survival, and metastatic ability of tumor cells, thereby promoting the occurrence and development of tumors. We used the tmb function and inferHeterogeneity function of the R software package maftools (version 2. 8. 05) to calculate the TMB and MATH of each tumor. At the same time, the MSI score of each tumor was obtained based on previous research^46^. Finally, we analyzed the correlation between TMB, MATH, and MSI with RAB3B gene expression.

### Promoter methylation level of RAB3B in pan-cancer.

Based on the UALCAN database (http://ualcan.path.uab.edu/index.html)^47^, we compared the DNA methylation levels of RAB3B between different types of cancer and their corresponding adjacent tissues. The goal is to gain a broader understanding of the relationship between DNA methylation levels of RAB3B in different tumor and normal samples. Students t-tests were used to evaluate statistical significance, p-values of 0.05 being considered statistically significant.

### Correlation analysis of RAB3B expression and immunological features.

We obtained a standardized pan-cancer dataset from the UCSC XENA database and extracted the gene expression profiles of each tumor. Using the TIMER^48^ algorithm implemented in the R package IOBR^49^, we evaluated the infiltration scores of B cells, T cells CD4, T cells CD8, Neutrophils, Macrophages, and Dendritic Cells in each tumor for every patient. Afterwards, we obtained the expression data for RAB3B and 60 genes that serve as markers for immune checkpoints^50^ in every sample. We then computed the Pearson correlation between RAB3B and these 60 marker genes. In the end, we employed the ESTIMATE package^51^ to calculate immune scores for every patient in every tumor using gene expression data. Then, we performed a correlation analysis to examine the relationship between these scores and the expression of the RAB3B gene.

### Statistics analysis

R software was used for all statistical analyses in this study. Wilcoxon rank sum tests were used to compare RAB3B expression over tumors and normal tissues. The hazard ratio (HR) and p-value were computed by conducting survival analysis using univariable Cox regression analysis. Pearson correlation analysis was employed. Some of the data was visualized using the Sangerbox (http://sangerbox.com/) and Xiantao (https://www.xiantaozi.com/) online tools. Statistical differences were considered significant if the p-value was below 0.05.

## Result

### The expression and ROC analysis of RAB3B in pan-cancer

**Figure 2:**
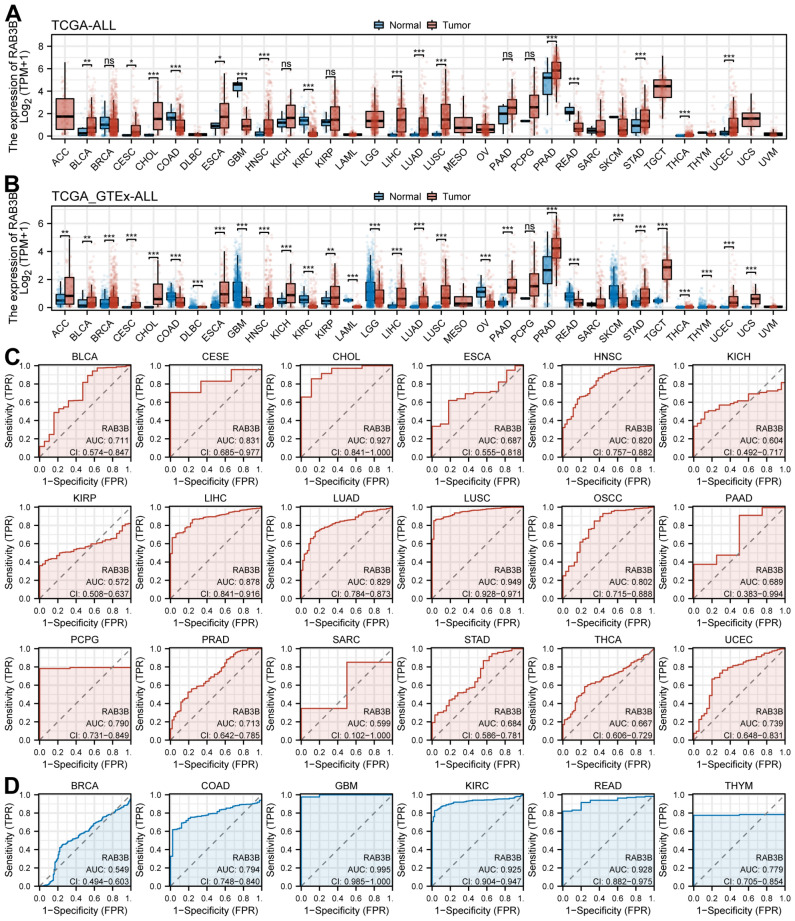
The expression and ROC analysis of RAB3B in pan-cancer. (**A**) Expression levels of RAB3B in pan-cancer were obtained from the TCGA database. (**B**) TCGA and GTEx databases were used to compare the expression differences of RAB3B between tumor and normal tissues in pan-cancer. (**C**,**D**) ROC curves for RAB3B in pan-cancer were generated. (*P < 0.05, **P < 0.01, ***P < 0.001. ns, not significant).

Through the analysis of the gene expression profiles of 33 tumors in the TCGA database, it was discovered that the expression levels of RAB3B exhibited notable disparities among different tumor samples and normal samples. With the exception of cancers lacking normal tissue data or having very limited normal samples, the expression of RAB3B in 16 cancers was found to be markedly distinct from that in normal tissues. The expression of RAB3B is increased in tumor samples of Bladder Urothelial Carcinoma (BLCA), Cervical squamous cell carcinoma and endocervical adenocarcinoma (CESC), Cholangiocarcinoma (CHOL), Esophageal carcinoma (ESCA), Head and Neck squamous cell carcinoma (HNSC), Liver hepatocellular carcinoma (LIHC), Lung adenocarcinoma (LUAD), Lung squamous cell carcinoma (LUSC), Prostate adenocarcinoma (PRAD), Stomach adenocarcinoma (STAD), Thyroid carcinoma (THCA), and Uterine Corpus Endometrial Carcinoma (UCEC) as shown in Fig. 2A.On the other hand, normal tissue samples of Colon adenocarcinoma (COAD), Glioblastoma multiforme (GBM), Kidney renal clear cell carcinoma (KIRC), and Rectum adenocarcinoma (READ) exhibited an increase in RAB3B expression. Furthermore, by conducting a collaborative examination of the GTEx database's data on samples from healthy individuals, we have identified a notable disparity in the expression of RAB3B between cancerous and healthy tissues across 29 different cancer types. Figure 2B demonstrates the upregulation of RAB3B in tumor samples of Adrenocortical carcinoma (ACC), BLCA, Breast invasive carcinoma (BRCA), CESC, CHOL, Lymphoid Neoplasm Diffuse Large B-cell Lymphoma (DLBC), ESCA, HNSC, Kidney Chromophobe (KICH), Kidney renal papillary cell carcinoma (KIRP), LIHC, LUAD, LUSC, Pancreatic adenocarcinoma (PAAD), PRAD, STAD, Testicular Germ Cell Tumors (TGCT), THCA, Thymoma (THYM), UCEC, and Uterine Carcinosarcoma (UCS). Conversely, it was found to be upregulated in normal tissue samples of COAD, GBM, KIRC, Acute Myeloid Leukemia (LAML), Brain Lower Grade Glioma (LGG), Ovarian serous cystadenocarcinoma (OV), READ, and Skin Cutaneous Melanoma (SKCM), which aligns with the findings from the TCGA database. These findings suggest that abnormal expression of RAB3B may be an important cause of tumor progression in many cancers. Afterwards, we generated ROC curves for each type of cancer at the pan-cancer level. The findings indicated that RAB3B exhibits a specific level of accuracy in diagnosing tumor samples in 11 datasets (Fig. 2C, AUC > 0.7) and normal samples in five datasets (Fig. 2D, AUC > 0.7). These datasets include BLCA (AUC = 0.711), CESC (AUC = 0.831), CHOL (AUC = 0.927), COAD (AUC = 0.794), HNSC (AUC = 0.820), GBM (AUC = 0.995), LIHC (AUC = 0.878), LUAD (AUC = 0.829), LUSC (AUC = 0.949), OSCC (AUC = 0.802), KIRC (AUC = 0.925), PCPG (AUC = 0.790), PRAD (AUC = 0.713), READ (AUC = 0.928), THYM (AUC = 0.779), and UCEC (AUC = 0.739).

### CPTAC analysis of RAB3B

**Figure 3:**
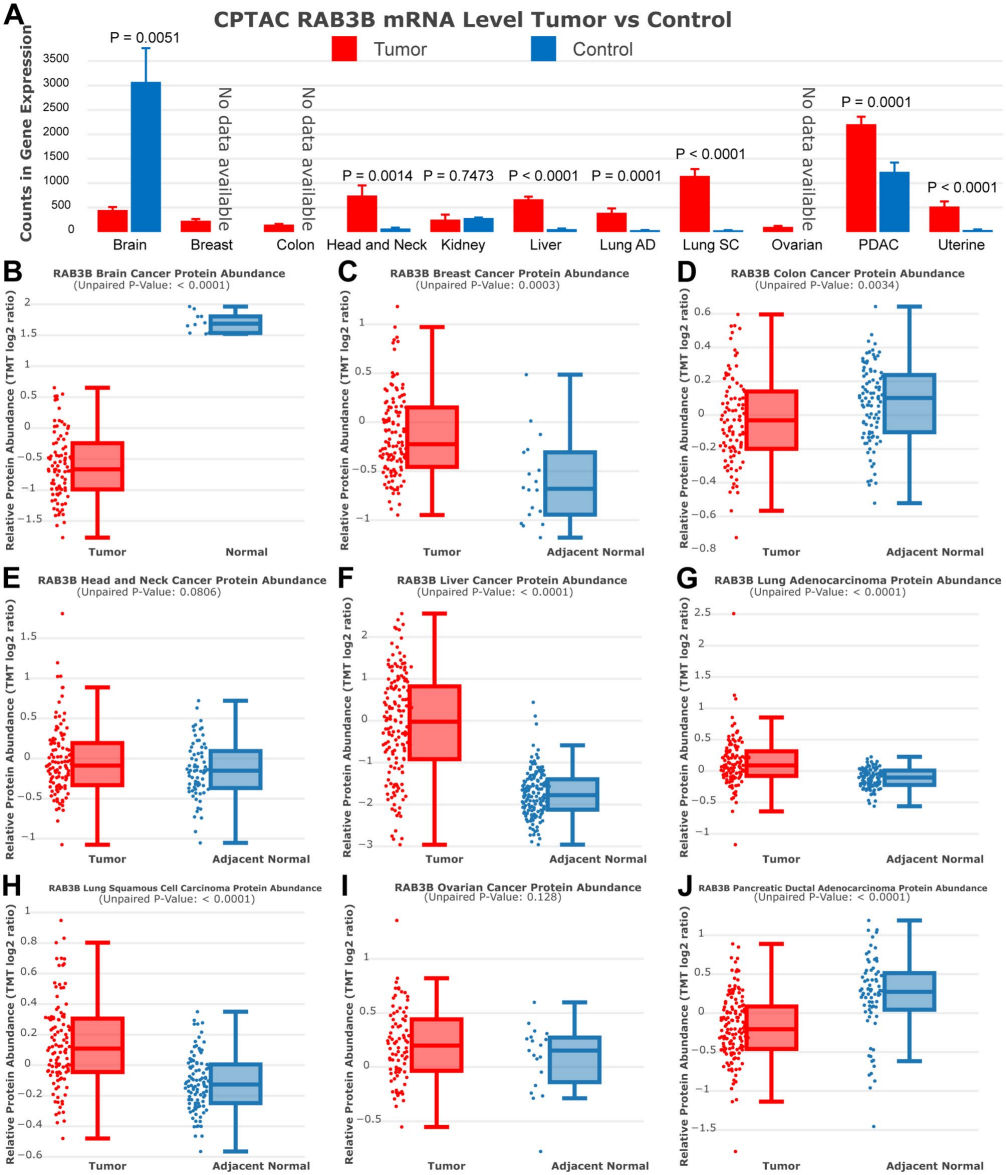
CPTAC analysis of RAB3B. (**A**) The CPTAC database analyzed differences in RNA expression of RAB3B in some cancers. (**B**–**J**) The CPTAC database analyzed the protein expression differences of RAB3B in some cancers.

We investigated the expression differences of RAB3B gene and protein in selected cancers by analyzing the CPTAC database. Utilizing the cProSite visualization tool, we observed a significant downregulation of RAB3B gene expression in brain tumor samples compared to normal tissues. Conversely, RAB3B gene expression was significantly upregulated in tumor samples of HNSC, LIHC, LUAD, LUSC, PDAC, and Uterine Cancer compared to normal samples. Protein expression analysis revealed a significant decrease in RAB3B protein expression levels in brain tumor, COAD and PDAC compared to normal samples. In contrast, the levels of RAB3B protein expression were notably elevated in tumor samples of BRCA, LIHC, LUAD, and LUSC when compared to the normal controls (Fig. 3).

### Protein expression scores from the human protein atlas

**Figure 4:**
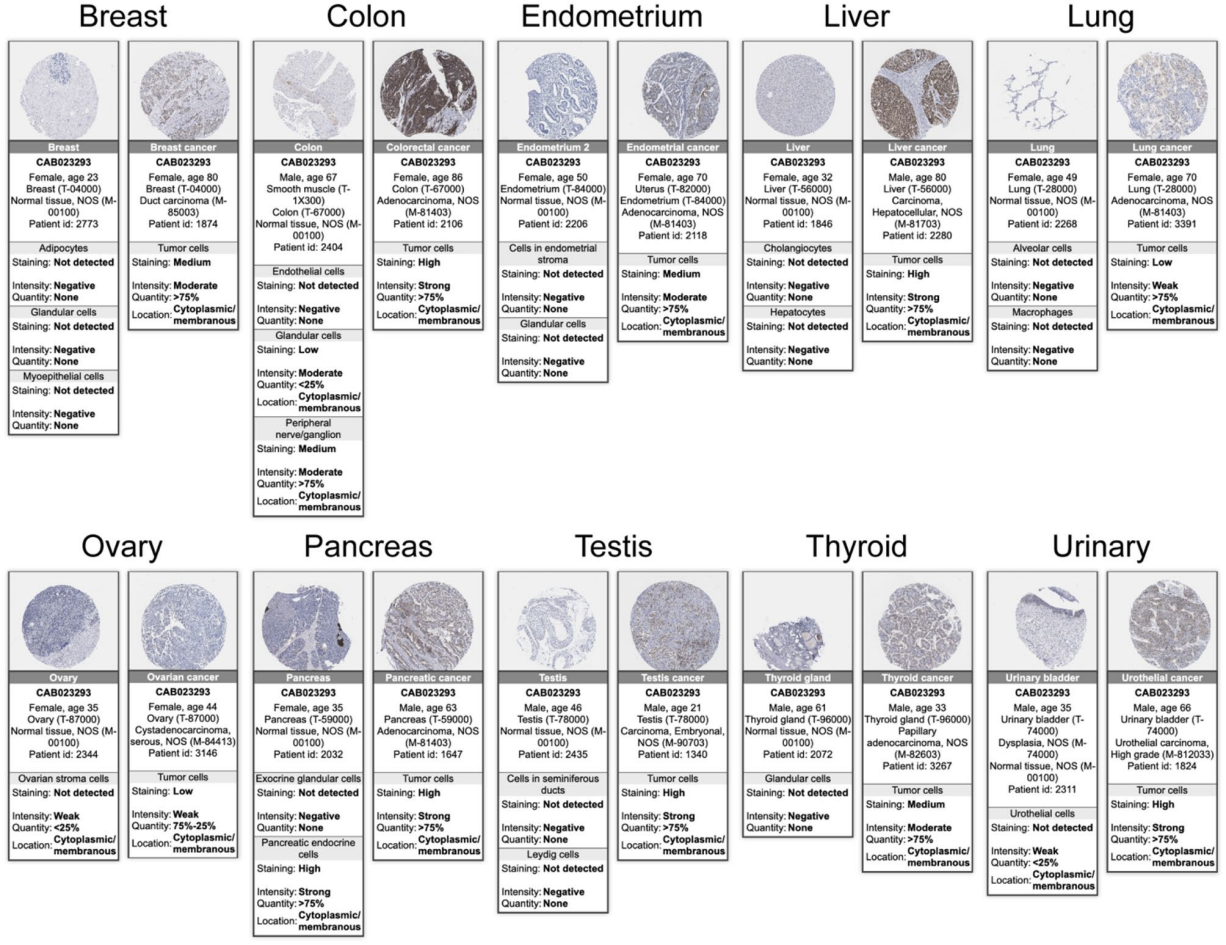
Protein expression scores from the Human Protein Atlas. The protein expression of RAB3B was visually analyzed through immunohistochemical images from both normal and tumor groups.

Furthermore, in order to accurately assess the protein expression of RAB3B, we utilized the Human Protein Atlas database to obtain immunohistochemical images. As depicted in Fig. 4, it is evident that RAB3B protein expression is significantly elevated in 10 types of cancer when compared to normal tissues. The provided data offers valuable understanding of the involvement of RAB3B in the development of cancer and holds significant implications for the creation of innovative treatment approaches. The precision and reliability of these findings offer an important contribution to the current body of research on RAB3B and its potential as a viable therapeutic target for cancer treatment.

### Confirmation of RAB3B expression in TMA

**Figure 5:**
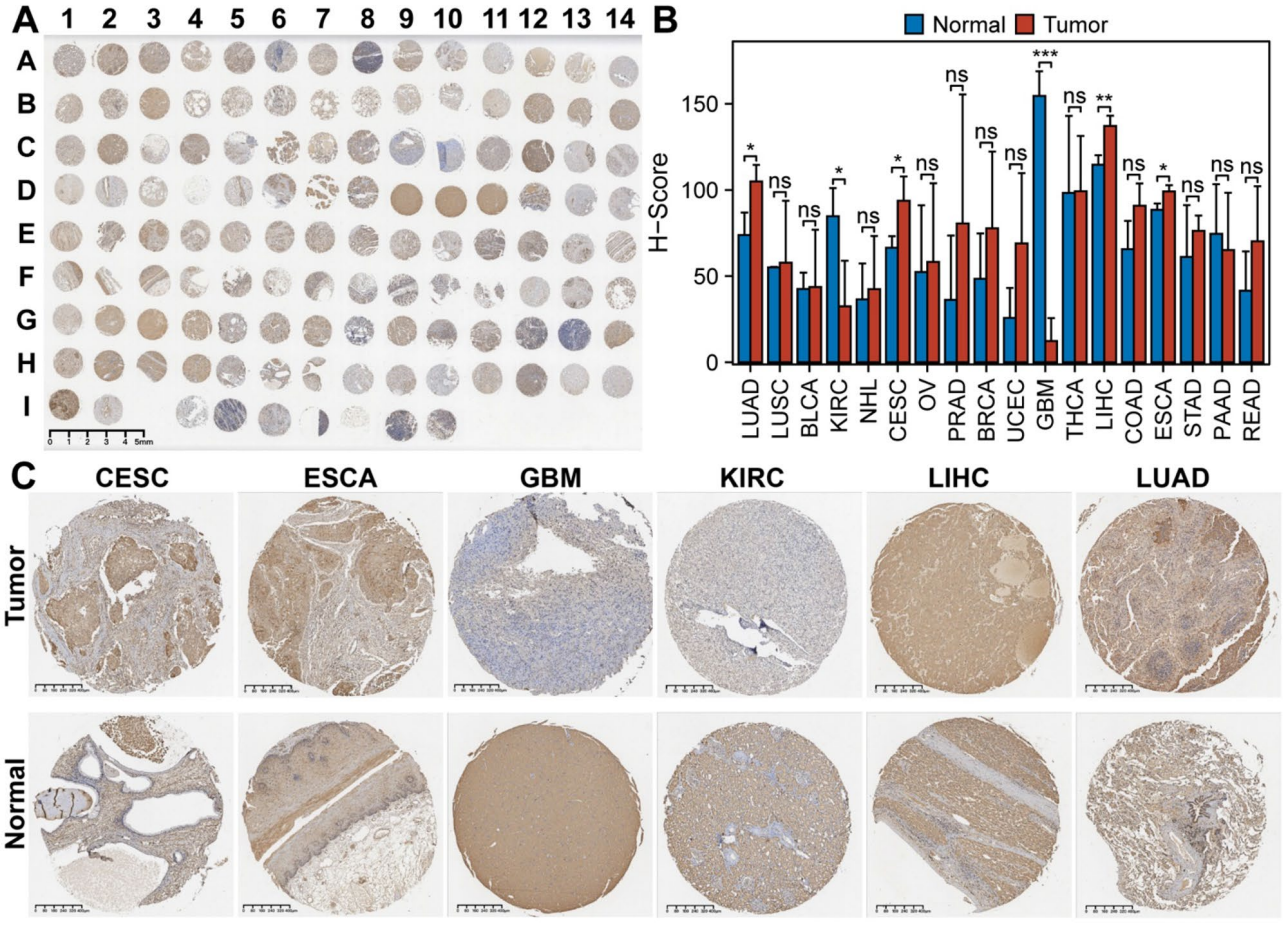
Confirmation of RAB3B expression in TMA. (**A**-**B**) Tissue microarrays of RAB3B protein expressed in 20 cancers. (**C**) Representative images of RAB3B protein expression in LUAD, CESC, KIRC, LIHC, ESCA and GBM.

To further validate the expression of RAB3B in different cancers, we selected TMA and used IHC staining to assess the expression of RAB3B (Fig. 5A). The IHC staining of tumor tissues indicated that RAB3B protein is mainly expressed in the cell membrane. Moreover, as there are only two samples of small cell lung cancer, and one malignant melanoma tissue was lost during processing, the above tumor samples were not sufficient for three cases, thus, the subsequent analysis did not include these two tumors. In the analysis of the other 18 tumors, we found that compared with the control group, the expression of RAB3B in LUAD, CESC, LIHC, and ESCA increased, while in KIRC and GBM, the expression of RAB3B decreased (Fig. 5B). These results are consistent with the expression of RAB3B in the pan-cancer analysis using TCGA and GTEx databases. Figure 5C is the representative image of the expression of RAB3B in malignant tumor tissues and control group tissues.

### Exploring the relationship between RAB3B and clinical features

**Figure 6:**
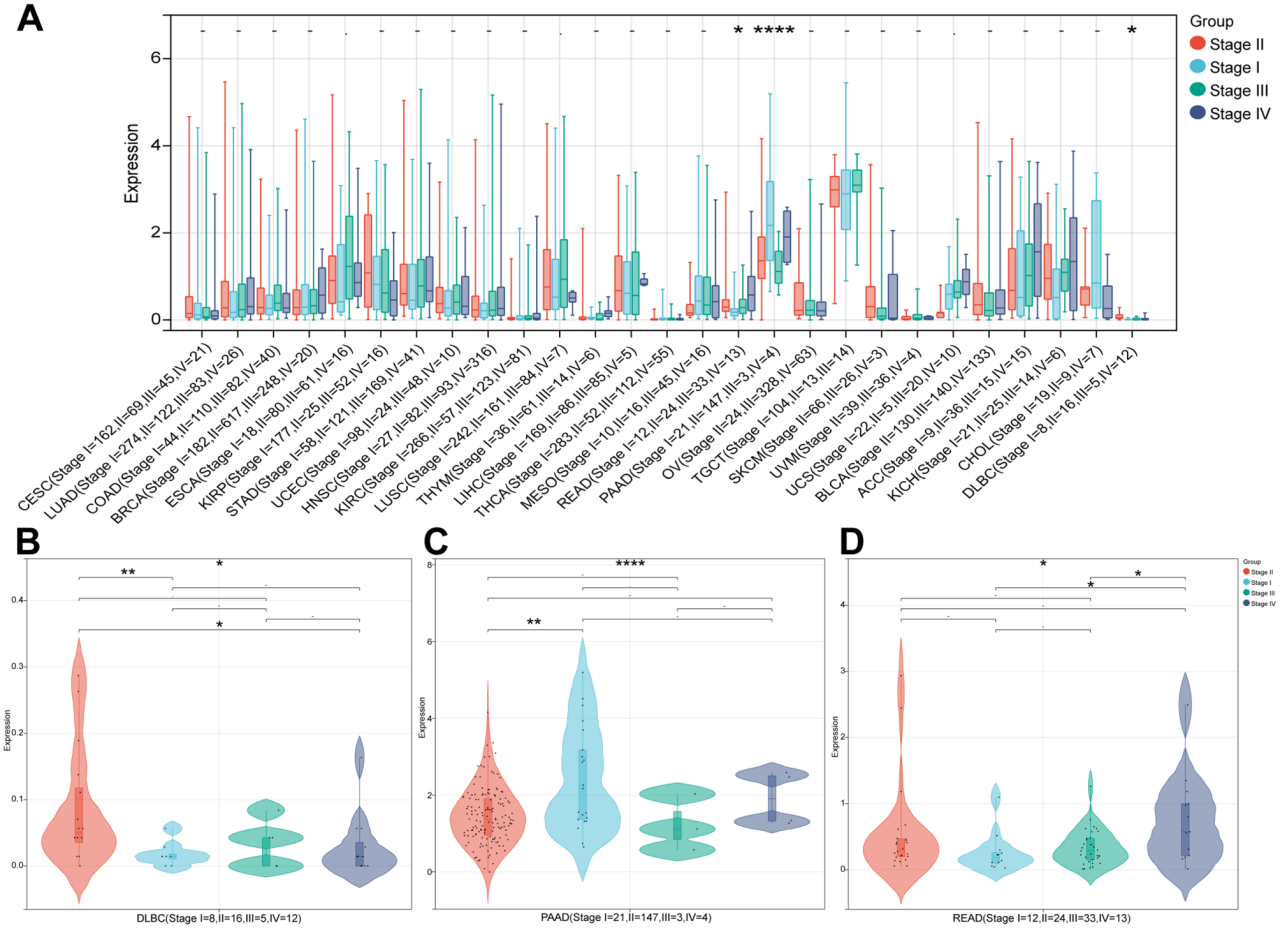
Exploring the relationship between RAB3B and clinical features. (**A**) RAB3B expression levels at distinct stages of pan-cancer. (**B**-**D**) It has been shown that RAB3B is expressed at different stages of cancer in DLBC, PAAD, and READ.

According to the perspective of pan-cancer, a thorough evaluation was conducted on the expression levels of RAB3B at different clinical and pathological stages. The findings indicated notable variations in the levels of RAB3B expression among three tumor types, namely DLBC, PAAD, and READ (Fig. 6A). The comprehensive statistical charts are displayed in Fig. 6B–D. It is important to note that while statistical significance was not observed in other tumors like LUAD, BRCA, THYM, LIHC, READ, UCS, ACC, and KICH, there was a tendency suggesting a substantial rise in RAB3B expression levels in advanced tumors, which somewhat emphasizes its prognostic significance. The discovery is beneficial for a comprehensive examination of the functioning of RAB3B and serves as a significant point of reference in comprehending the progression of different types of tumors.

### Pan-cancer prognostic assessment of RAB3B

**Figure 7:**
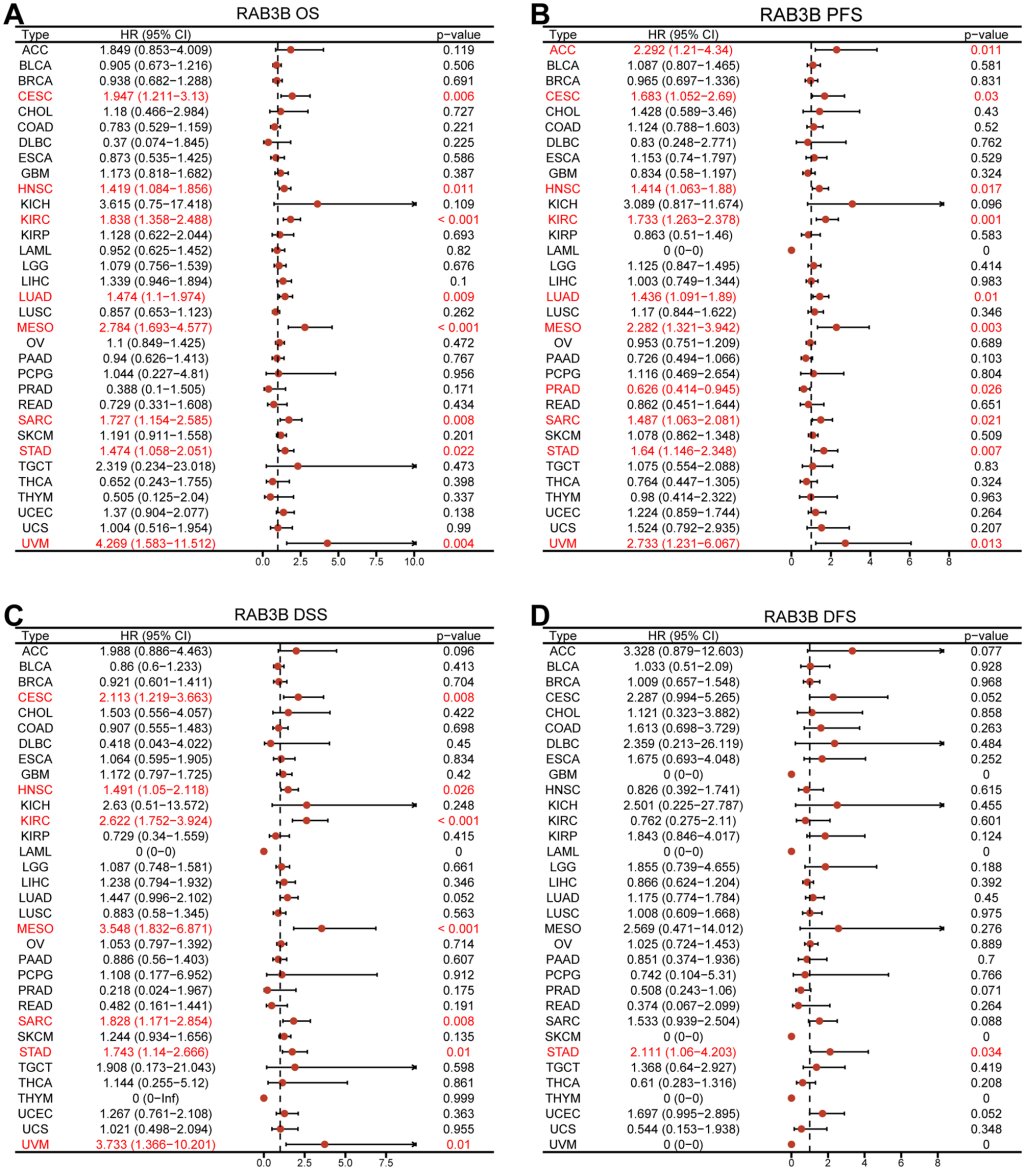
Pan-cancer prognostic assessment of RAB3B. (**A**) Correlation between RAB3B expression and OS; (**B**) PFS; (**C**) DSS; (**D**) DFS. OS, overall survival; PFS, progression-free survival; DSS, disease-specific survival; DFS, disease-free survival.

The survival analysis of RAB3B suggests its significant prognostic value in the field of oncology. Through employing the sophisticated technique of Cox regression analysis, it has been established that elevated levels of RAB3B serve as detrimental risk factors influencing overall survival in numerous tumor classifications such as CESC, HNSC, KIRC, LUAD, MESO, SARC, STAD, and UVM, thus elucidating its significance as a potent biomarker within cancer scientific inquiry (Fig. 7A). Examination across distinct malignancy classes reveals a robust connection between heightened RAB3B expression and PFS, notably within subtypes like ACC, CESC, HNSC, KIRC, LUAD, MESO, PRAD, SARC, STAD, and UVM (Fig. 7B). Furthermore, additional scrutiny disclosed that elevated RAB3B mRNA is correlates with reduced DSS in malignancies such as CESC, HNSC, KIRC, MESO, SARC, STAD, and UVM (Fig. 7C). Refinement through univariate version of Cox regression analysis indicated a notable link between elevated RAB3B expression and unfavorable prognostics in STAD (Fig. 7D). In closing, these findings support the notion that RAB3B may hold considerable prognostic worth in cancer and provide beneficial guidance for actual medical treatments.

### Pan-cancer analysis of RAB3B gene changes

**Figure 8:**
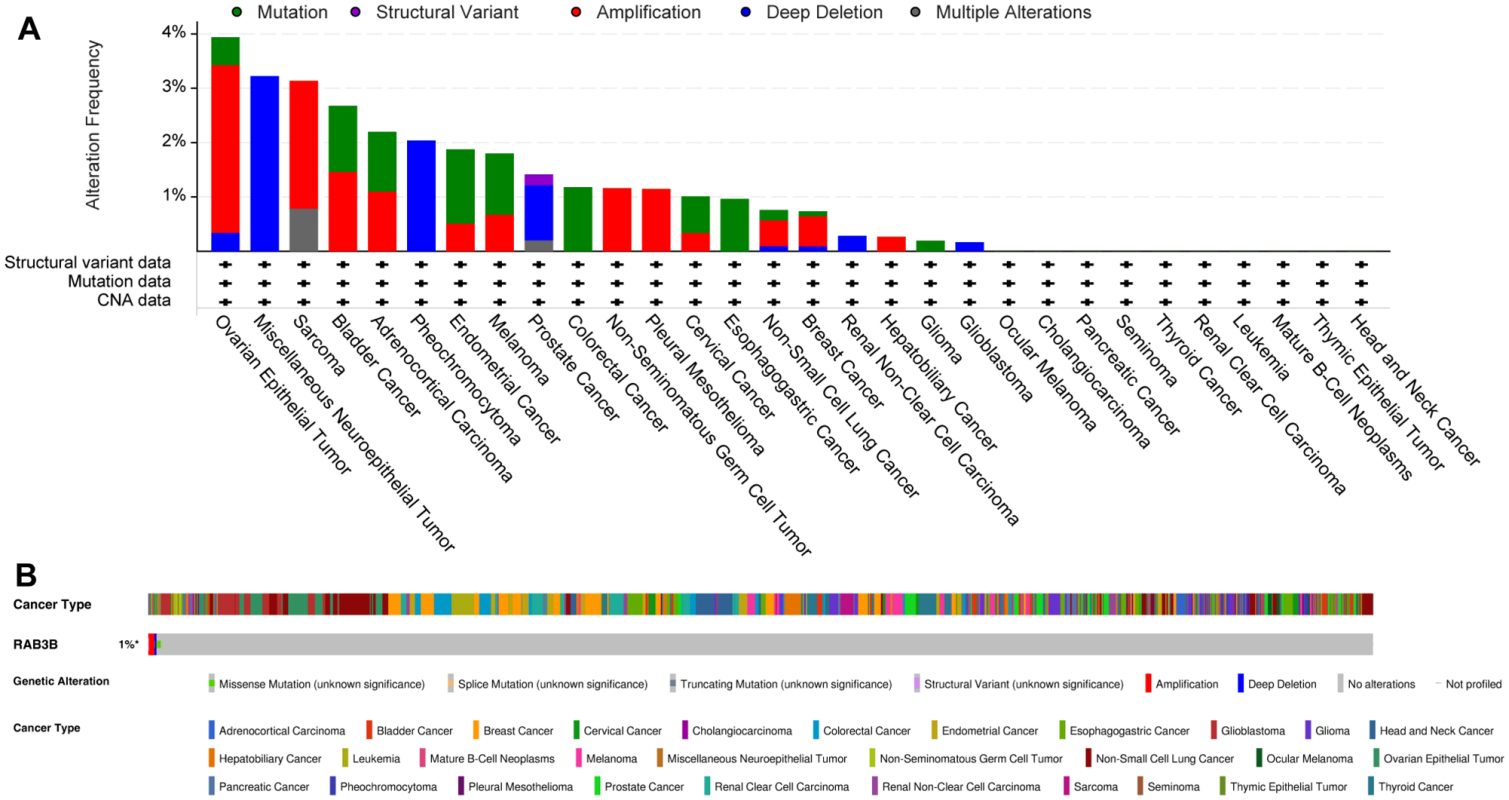
Pan-cancer analysis of RAB3B gene changes. (**A**) Alteration frequency of RAB3B. (**B**) From cBioPortal, an OncoPrint visual summary is provided of the alterations found in a query of RAB3B.

A total of 10,953 patients from the TCGA database were analyzed using the cBioPortal platform to determine whether the RAB3B gene was mutated in tumor tissues. The largest percentage of all mutation types was attributed to the amplification of RAB3B, with Deep Deletion following closely behind. Among them, the mutation rates of Ovarian Epithelial Tumor, Miscellaneous Neuroepithelial Tumor, and Sarcoma are the highest, with 3.94%, 3.23%, and 3.14%, respectively. Interestingly, in Miscellaneous Neuroepithelial Tumor, the mutation type of RAB3B is Deep Deletion (Fig. 8A,B).

### Genomic heterogeneity and gene expression analysis

**Figure 9:**
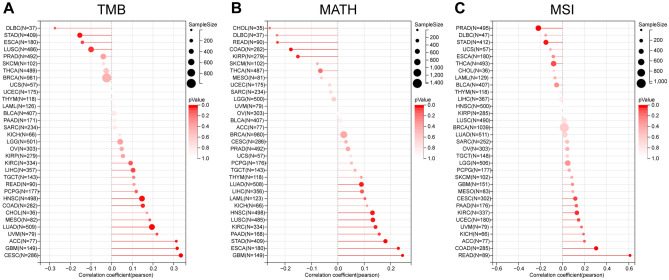
Genomic heterogeneity and gene expression analysis. (A) An analysis of the correlations between RAB3B expression and TMB in pan-cancer. (B) An analysis of the correlations between RAB3B expression and MATH in pan-cancer. (C) An analysis of the correlations between RAB3B expression and MSI in pan-cancer.

Observing GBM, CESC, LUAD, COAD, HNSC, ACC, we find RAB3B's expression positively tied to TMB but inversely related to STAD and LUSC (Fig. 9A). Notably, in several types like GBM, LUAD, ESCA, STAD, HNSC, KIRC, LUSC, and PAAD, RAB3B displays a positive correlation with MATH. Interestingly though, this relationship trends negative with regard to MATH in COAD, KIRP, and READ (Fig. 9B). Additionally, RAB3B's presence in CESC, COAD, KIRC, and READ correlates positively to MSI, yet negatively in STAD and PRAD (Fig. 9C).

### Promoter methylation level of RAB3B in pan-cancer

**Figure 10:**
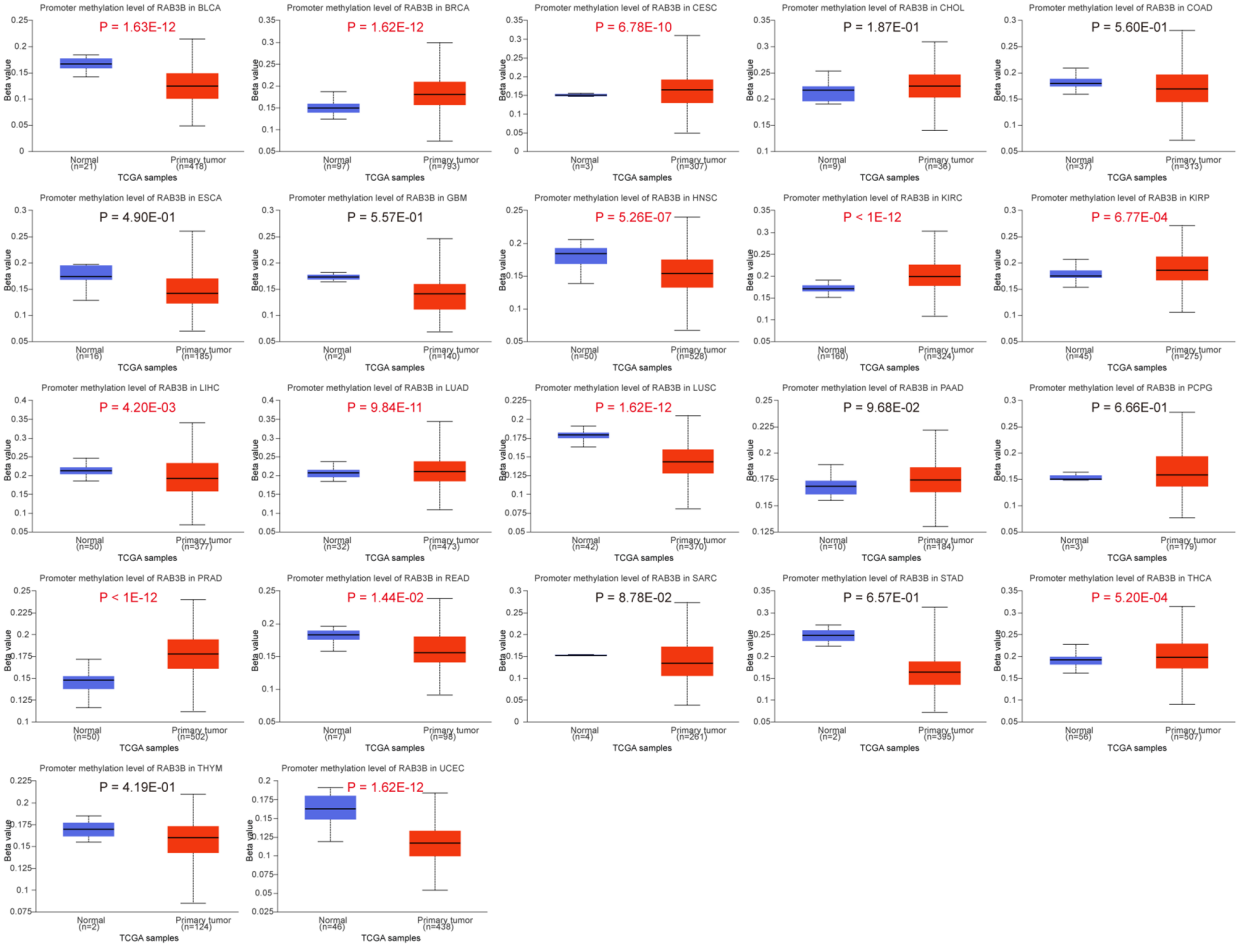
Promoter methylation level of RAB3B in pan-cancer.

UALCAN database analysis showed lower RAB3B DNA methylation levels in BLCA, HNSC, LIHC, LUSC, READ, and UCEC cancerous tissues when compared to healthy ones. By contrast, higher RAB3B methylation occurred in BRCA, CESC, KIRC, KIRP, LUAD, PRAD, and THCA tumor samples. Notably, CHOL, COAD, ESCA, GBM, PAAD, PCPG, SARC, STAD, and THYM tumor/normal tissue pairs showed no significant methylation difference (Fig. 10).

### Correlation analysis of RAB3B expression with immune characteristics

**Figure 11:**
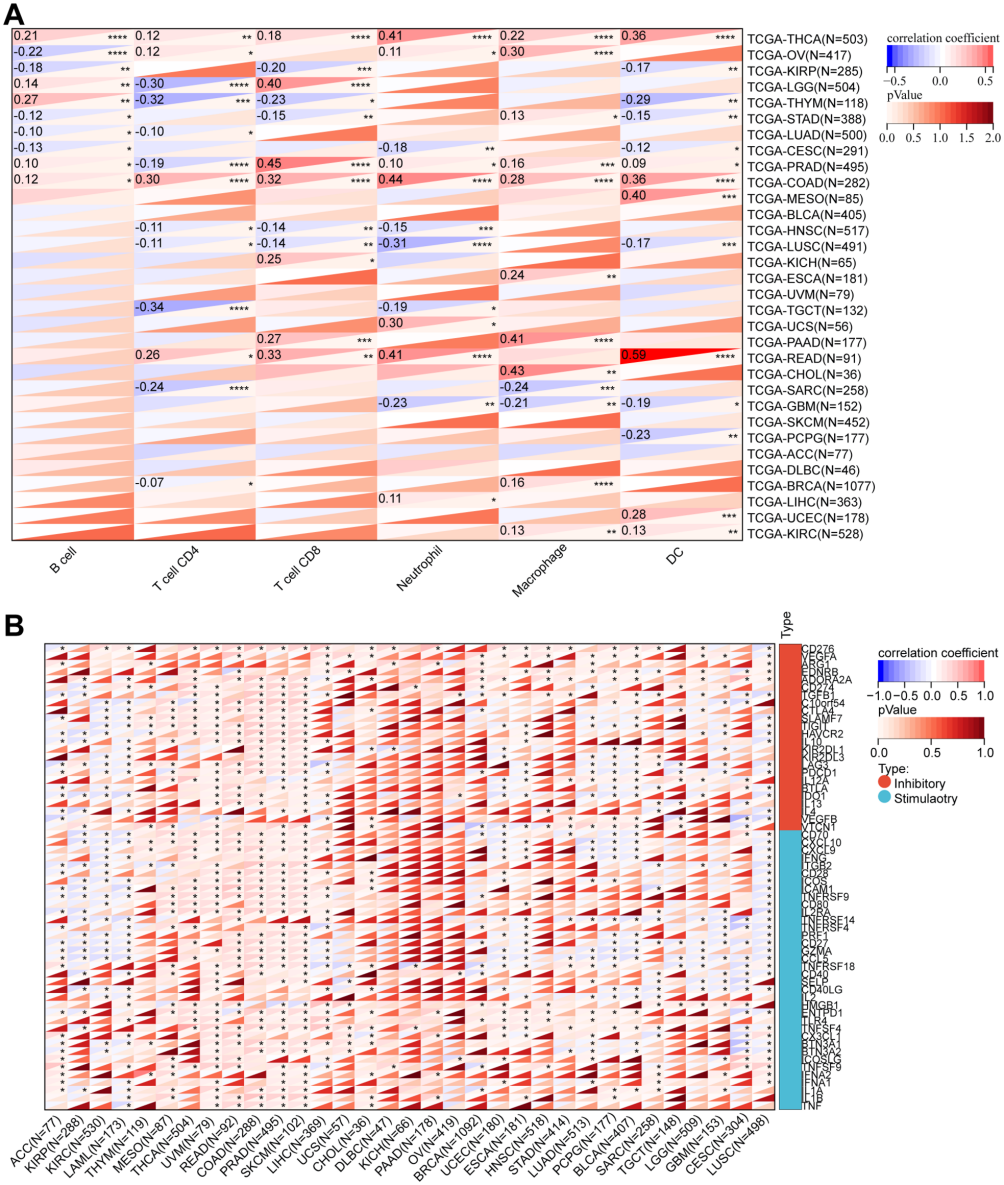
Correlation of RAB3B with the level of immune infiltrating cells and immune checkpoint. (**A**) The level of immune infiltrating cells is correlated with RAB3B. (**B**) Correlation of RAB3B with the immune checkpoint.

Our study examined the relationship between RAB3B expression and immune cell infiltration in 32 types of tumors. Infiltration scores were calculated for six immune cell categories and correlated with RAB3B expression. In 29 out of 32 cancer types, we observed a significant correlation between RAB3B expression and immune infiltration (Fig. 11A). These cancer types include BRCA, CESC, CHOL, COAD, ESCA, GBM, HNSC, KICH, KIRC, KIRP, LGG, LIHC, LUAD, LUSC, MESO, OV, PAAD, PCPG, PRAD, READ, SARC, STAD, TGCT, THCA, THYM, UCEC, and UCS. It is interesting to note that RAB3B expression showed a positive correlation with immune cell infiltration across all six categories of COAD and THCA.

We also investigated the relationship between RAB3B expression and 60 immune checkpoint pathway genes in pan-cancer. As shown in Fig. 11B, RAB3B exhibited significant associations with immune inhibitory/stimulatory genes listed in pan-cancer. Specifically, in MESO, THCA, UVM, READ, COAD, PRAD, SKCM, and LIHC, RAB3B correlated positively with most of the immune checkpoint genes. In contrast, RAB3B correlated negatively with most immune checkpoint genes in ACC, KIRP, KIRC, LAML, UCEC, ESCA, LUAD, PCPG, BLCA, SARC, LGG, GBM, CESC, and LUSC. Further investigation revealed a significant correlation between CD276 and RAB3B in 22 tumor types, highlighting the need for further mechanistic studies.

**Figure 12:**
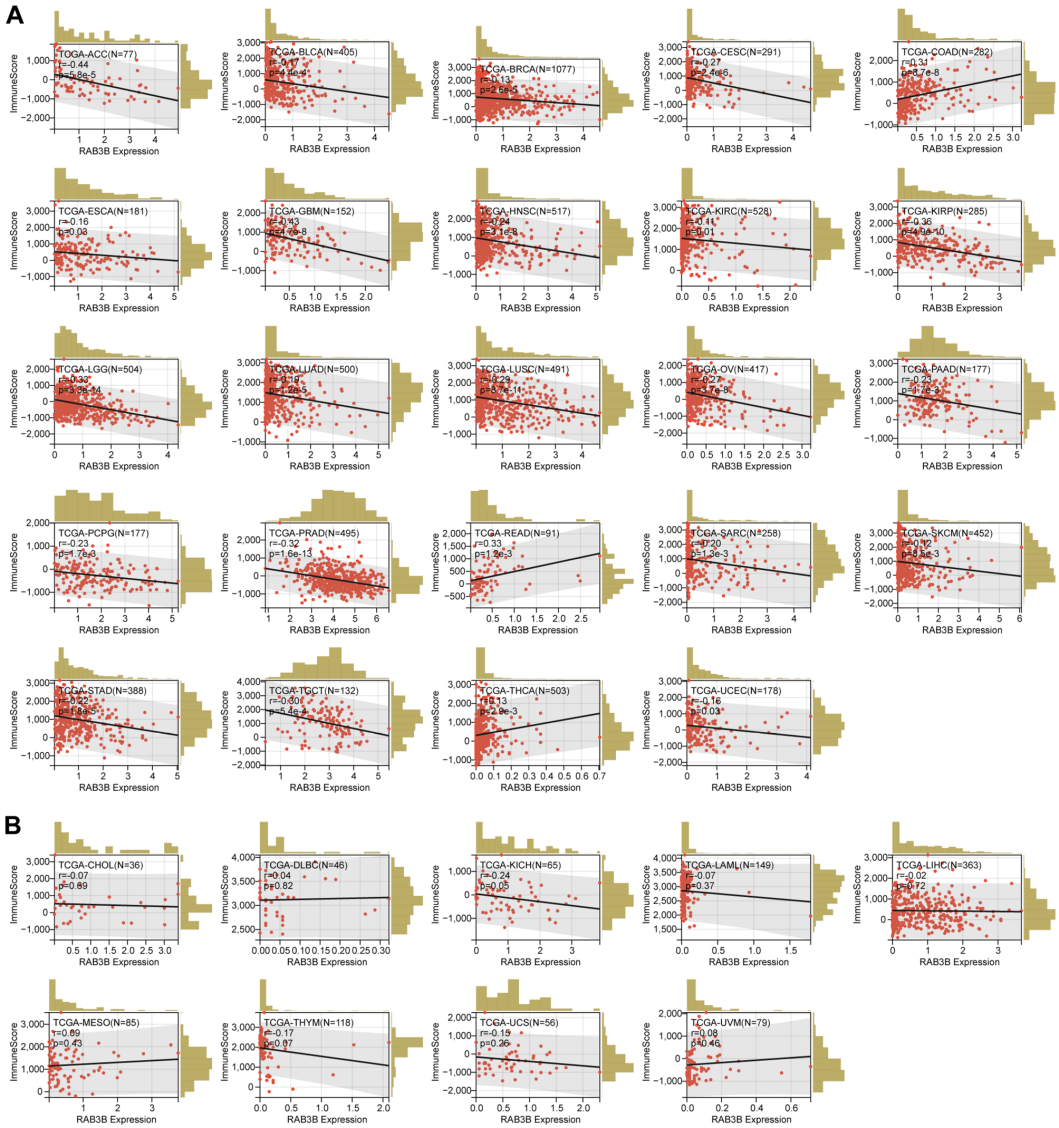
Correlation of RAB3B with the ImmuneScore in pan-cancer. (**A**) RAB3B has a significant correlation with ImmuneScore in 24 types of tumors. (**B**) The correlation between RAB3B and the ImmuneScore of nine different tumors was found to be non-significant.

In addition, we investigated how RAB3B correlates with pan-cancer immune scores in the tumor microenvironment (TME). In COAD, THCA, and READ, there was a notable and favorable association observed between the expression of RAB3B and ImmuneScore, as indicated by our findings in Fig. 12A. It was found, however, that RAB3B expression correlated negatively with ImmuneScore in ACC, BLCA, BRCA, CESC, ESCA, GBM, HNSC, KIRC, KIRP, LGG, LUAD, LUSC, OV, PAAD, PCPG, PRAD, SARC, SKCM, STAD, TGCT, and UCEC (Fig. 12A). As shown in Fig. 12B, RAB3B expression and ImmuneScore did not correlate significantly in CHOL, DLBC, KICH, LAML, LIHC, MESO, THYM, UCS, and UVM. These findings highlight the importance of further investigating the role of RAB3B in the TME.

## Discussion

Cancer is a complex disease and remains an important global health challenge^1,52,53^. Significant progress has been made in cancer research, but there are still many unknown areas that require further investigation^2,54^. Currently, the primary focuses of cancer investigation encompass causation, mitigation, identification, and therapy. The pan-cancer analysis is a valuable approach that aims to uncover resemblances and variations among various types of cancers, offering profound perspectives for the development of strategies for cancer prevention and personalized treatment^2,55–60^. Recent research has placed more emphasis on comprehensive analysis of the entire genome in order to uncover gene mutations, alterations in methylation, and variations in the immune microenvironment linked to the onset and progression of cancer. This holds immense importance in terms of early detection and identification of responsive biomarkers.

The RAB protein family is a group of proteins that participate in cell membrane transport and play important roles in the intracellular transport process^23,61^. RAB3B, a member of the Rab protein family, has been found to be associated with various cancers^25–35,62^. Luo et al.^25^ discovered that the level of RAB3B expression was associated with the advancement of gliomas. Additionally, the inhibition of RAB3B greatly impeded cell proliferation in gliomas by halting the cell cycle and triggering apoptosis. Ye et al.^27^ found that the inhibition of breast cancer cell proliferation and invasion was observed when RAB3B was knocked down. Moreover, research has additionally discovered that RAB3B is excessively expressed in gliomas, prostate carcinoma, lung adenocarcinoma, and gastric carcinoma. Additionally, individuals with elevated levels of expression have a more unfavorable prognosis^30–34^. However, these studies on RAB3B in cancer are limited to some extent. Hence, additional investigation on the manifestation of RAB3B in comprehensive cancer datasets and its possible links to tumor diversity, alterations in methylation, and the immune microenvironment may offer novel avenues for clinical cancer therapy.

By analyzing TCGA and GTEx databases, comprehensive analyses of RAB3B expression in pan-cancer datasets were performed. The findings indicated that the levels of RAB3B were notably elevated in ACC, BLCA, BRCA, CESC, CHOL, DLBC, ESCA, HNSC, KICH, KIRP, LIHC, LUAD, LUSC, PAAD, PRAD, STAD, TGCT, THCA, THYM, UCEC, and UCS than in controls, while its expression was significantly lower in COAD, GBM, KIRC, LAML, LGG, OV, READ, and SKCM. Further examination of the CPTAC and HPA databases confirmed the variations in protein expression of RAB3B across multiple cancer types. At the same time, we confirmed the expression of RAB3B in TMAs. The IHC results showed that the expression of RAB3B was consistent with the results of online database analysis in six types of tumors, including LUAD, CESC, KIRC, LIHC, ESCA and GBM. These results suggest that RAB3B displays different expression patterns in various cancers, indicating its importance in cancer development. The ROC curve is a valuable tool in assessing the precision and discriminatory power of cancer diagnostic tests, offering crucial insights for the timely detection and treatment of cancer^63,64^. As reported in this study, we found that the overexpression of RAB3B in 11 types of cancer was associated with a certain degree of diagnostic accuracy, especially in GBM, where the AUC value was close to 1, indicating its potential diagnostic value. Furthermore, using Cox regression analysis (including OS, PFS, DSS, and DFS), we investigated the association between expression levels of RAB3B and prognosis. In ACC, CESC, HNSC, KIRC, LUAD, MESO, SARC, STAD, and UVM, it was discovered that a strong correlation existed between elevated RAB3B levels and unfavorable prognosis. Conversely, patients with high RAB3B expression in PRAD exhibited a more favorable prognosis. The findings suggest that RAB3B may be associated with the progression of specific subtypes of cancer.

Using the cBioPortal platform for analyzing gene and expression data from various cancers, we found that RAB3B had mutations in most cancers, with the highest mutation rate in Ovarian Epithelial Tumor, Miscellaneous Neuroepithelial Tumor, and Sarcoma. Furthermore, we have also discovered a notable association between the expression of RAB3B and several cancer-related indicators, such as TMB, MATH, and MSI. These indices are closely related to gene mutations, which in turn can affect the proliferation, survival, and metastasis of tumor cells^10,65–67^. Therefore, alterations in RAB3B genes and atypical expression might have significant implications in the progression of different types of malignancies. Furthermore, we revealed the role of DNA methylation in tumors. Under normal circumstances, methylation regulates gene expression, but in tumors, abnormal methylation may lead to the inactivation of tumor suppressor genes and activation of oncogenes, promoting tumor development^12,13,68^. Our study found changes in the methylation of RAB3B in 13 types of cancer tissues, indicating the universality of abnormal methylation in various cancers. Compared with other relevant studies, our study revealed the multiple roles of RAB3B in cancer, not only at the levels of gene mutation and expression but also with regards to its influence on methylation. The findings from our research contribute to a better comprehension of the mechanisms involved in cancer progression and offer a theoretical foundation for the exploration of novel approaches to treatment.

Tumor development and incidence are closely related to immune cell infiltration^14,15^. We discovered a strong association between the expression of the RAB3B gene and the level of immune cell infiltration in various types of cancer, specifically in COAD and THCA. In these two types of cancer, RAB3B expression exhibited a positive correlation with all six categories of immune cell infiltration. To maintain the balance of the immune system, immune checkpoints serve as mechanisms that control immune responses^19,20,69^. This study discovered a notable association between RAB3B and presently listed genes that either suppress or enhance the immune response in various types of cancer. CD276 and RAB3B had a significant correlation in 22 tumors. Studies have shown that CD276, as an immune checkpoint, enables cancer stem cells to evade immune surveillance. Blocking CD276 can effectively enhance T cell-mediated antitumor immunity, eliminate cancer stem cells, and prevent tumor growth and metastasis^70^. The potential relationship between RAB3B and CD276 can act as a unique potential target for cancer immunotherapy. Tumor tissues are assessed for immune cell infiltration using the immune score. The findings from our study indicated a strong positive association between the expression of RAB3B and ImmuneScore in COAD, THCA, and READ. These results align with the outcomes of TIMER analysis, providing additional confirmation of its involvement in immune response.

## Conclusion

Based on our pan-cancer analysis, we found significant differences in RAB3B expression between most cancerous and normal tissues, and a correlation between RAB3B expression and clinical outcome. We found that high expression of RAB3B is moderately accurate in diagnosing most cancers, and may serve as an independent prognostic factor in many cancers. In addition, we have identified mutations in RAB3B in most cancers, and its expression levels are significantly associated with cancer-related indicators such as TMB, MATH, and MSI. Most cancers exhibit significant DNA methylation abnormalities compared to normal tissues. There is also a significant correlation between RAB3B expression and immune scores in various types of cancers, as well as a significant positive correlation between RAB3B expression and CD276 in 22 types of cancer. Our analysis of a larger sample size has yielded more comprehensive insights into the role of RAB3B in the mechanisms of cancer development, surpassing previous studies. The next step will be to collect more samples and perform more detailed analyses to better understand the mechanisms of RAB3B in cancer.

## Data Availability

The datasets generated during and/or analysed during the current study are available from the corresponding author on reasonable request.

## References

[1] Sung H, Ferlay J, Siegel RL, Laversanne M, Soerjomataram I, Jemal A, Bray F (2021). Global cancer statistics 2020: GLOBOCAN estimates of incidence and mortality worldwide for 36 cancers in 185 countries. CA Cancer J. Clin..

[2] Hoadley KA, Yau C, Hinoue T, Wolf DM, Lazar AJ, Drill E, Shen R, Taylor AM, Cherniack AD, Thorsson V (2018). Cell-of-origin patterns dominate the molecular classification of 10,000 tumors from 33 types of cancer. Cell.

[3] Ozga AJ, Chow MT, Luster AD (2021). Chemokines and the immune response to cancer. Immunity.

[4] Zhong H, Shi Q, Wen Q, Chen J, Li X, Ruan R, Zeng S, Dai X, Xiong J, Li L (2023). Pan-cancer analysis reveals potential of FAM110A as a prognostic and immunological biomarker in human cancer. Front. Immunol..

[5] Ma C, Pu W, Wang B, Gu B, Gao L, Wang N, Cui H, Chen H (2022). A pan-cancer analysis suggests an important role of *TMEM45A* in the immune response against stomach adenocarcinoma. J. Biol. Regul Homeost Agents.

[6] Jiang X, He J, Wang Y, Liu J, Li X, He X, Cai H (2022). A pan-cancer analysis of the biological function and clinical value of BTLA in tumors. Biocell.

[7] Chen W, Shan Y, Li J (2023). A pan-cancer analysis of deubiquitinating enzyme ubiquitin-specific protease 9X as a prognostic and immunological biomarker in human tumors. J. Biol. Reg. Homeos Agent.

[8] Cheng HH, Sokolova AO, Schaeffer EM, Small EJ, Higano CS (2019). Germline and somatic mutations in prostate cancer for the clinician. J. Natl. Compr. Cancer Netw..

[9] Khan AA, Liu X, Yan X, Tahir M, Ali S, Huang H (2021). An overview of genetic mutations and epigenetic signatures in the course of pancreatic cancer progression. Cancer Metast Rev..

[10] Rizzo A, Ricci AD, Brandi G (2021). PD-L1, TMB, MSI, and other predictors of response to immune checkpoint inhibitors in biliary tract cancer. Cancers.

[11] Ma D, Jiang Y-Z, Liu X-Y, Liu Y-R, Shao Z-M (2017). Clinical and molecular relevance of mutant-allele tumor heterogeneity in breast cancer. Breast Cancer Res. Treat..

[12] Esteller M (2007). Cancer epigenomics: DNA methylomes and histone-modification maps. Nat. Rev. Genet..

[13] Baylin SB, Jones PA (2016). Epigenetic Determinants of Cancer. Cold Spring Harbor Perspect Biol..

[14] Mao X, Xu J, Wang W, Liang C, Hua J, Liu J, Zhang B, Meng Q, Yu X, Shi S (2021). Crosstalk between cancer-associated fibroblasts and immune cells in the tumor microenvironment: New findings and future perspectives. Mol. Cancer.

[15] Dieci MV, Miglietta F, Guarneri V (2021). Immune infiltrates in breast cancer: recent updates and clinical implications. Cells.

[16] Xiao Y, Jiang J, Chen Y, Huang Y, Wei C (2022). PD-1 relevant genes predict the prognosis of breast cancer and their prediction effect in tumor response to immunotherapy. Oncologie.

[17] Liu X-S, Liu C, Zeng J, Zeng D-B, Chen Y-J, Tan F, Gao Y, Liu X-Y, Zhang Y, Zhang Y-H (2022). Nucleophosmin 1 is a prognostic marker of gastrointestinal cancer and is associated with m6A and cuproptosis. Front. Pharmacol..

[18] Liu X-S, Zhou L-M, Yuan L-L, Gao Y, Kui X-Y, Liu X-Y, Pei Z-J (2021). NPM1 Is a prognostic biomarker involved in immune infiltration of lung adenocarcinoma and associated With m6A modification and glycolysis. Front. Immunol..

[19] Topalian SL, Taube JM, Anders RA, Pardoll DM (2016). Mechanism-driven biomarkers to guide immune checkpoint blockade in cancer therapy. Nat. Rev. Cancer.

[20] Darvin P, Toor SM, Sasidharan Nair V, Elkord E (2018). Immune checkpoint inhibitors: recent progress and potential biomarkers. Exp. Mol. Med..

[21] Na KJ, Choi H, Oh HR, Kim YH, Lee SB, Jung YJ, Koh J, Park S, Lee HJ, Jeon YK (2020). Reciprocal change in Glucose metabolism of Cancer and Immune Cells mediated by different Glucose Transporters predicts Immunotherapy response. Theranostics.

[22] Gu J, Wang Z, Wang BO, Ma X (2023). ImmuneScore of eight-gene signature predicts prognosis and survival in patients with endometrial cancer. Front. Oncol..

[23] Guadagno NA, Progida C (2019). Rab GTPases: Switching to human diseases. Cells.

[24] Raffaniello RD (2021). Rab3 proteins and cancer: Exit strategies. J. Cell. Biochem..

[25] Luo Q, Liu Y, Yuan Z, Huang L, Diao B (2021). Expression of Rab3b in human glioma: Influence on cell proliferation and apoptosis. Curr. Pharm. Des..

[26] Tan PY, Chang CW, Chng KR, Wansa KDSA, Sung W-K, Cheung E (2012). Integration of regulatory networks by NKX3-1 promotes androgen-dependent prostate cancer survival. Mol. Cell. Biol..

[27] Ye F, Tang H, Liu Q, Xie X, Wu M, Liu X, Chen B, Xie X (2014). miR-200b as a prognostic factor in breast cancer targets multiple members of RAB family. J. Transl. Med..

[28] Klengel R, Piiper A, Pittelkow S, Zeuzem S (1997). Differential expression of rab3 isoforms during differentiation of pancreatic acinar cell Line AR42J. Biochem. Biophys. Res. Commun..

[29] Tsunedomi R, Yoshimura K, Kimura Y, Nishiyama M, Fujiwara N, Matsukuma S, Kanekiyo S, Matsui H, Shindo Y, Watanabe Y (2022). Elevated expression of RAB3B plays important roles in chemoresistance and metastatic potential of hepatoma cells. BMC Cancer.

[30] Zhang C, Min L, Zhang L, Ma Y, Yang Y, Shou C (2016). Combined analysis identifies six genes correlated with augmented malignancy from non-small cell to small cell lung cancer. Tumor Biol..

[31] Liu Q, Tang H, Liu X, Liao Y, Li H, Zhao Z, Yuan X, Jiang W (2014). miR-200b as a prognostic factor targets multiple members of RAB family in glioma. Med. Oncol..

[32] Wang L, Skotland T, Berge V, Sandvig K, Llorente A (2017). Exosomal proteins as prostate cancer biomarkers in urine: From mass spectrometry discovery to immunoassay-based validation. Eur. J. Pharm. Sci..

[33] Li C, Zou X, Cai Q, Li J, Yang S, Zhang A, Chen C, Zhu L (2022). Comprehensive expression profile analysis of neutrophil extracellular trap-affected genes in gastric cancer cells and the clinical significance of lncRNA NEAT1-related signaling. Front. Oncol..

[34] Zeng Z, Xie D, Gong J (2020). Genome-wide identification of CpG island methylator phenotype related gene signature as a novel prognostic biomarker of gastric cancer. PeerJ.

[35] Kottorou A, Dimitrakopoulos F-I, Diamantopoulou G, Kalofonou F, Stavropoulos M, Thomopoulos K, Makatsoris T, Koutras A, Kalofonos H (2023). Small extracellular vesicles (sEVs) biogenesis molecular players are associated with clinical outcome of colorectal cancer patients. Cancers.

[36] Tomczak K, Czerwińska P, Wiznerowicz M (2015). Review The Cancer Genome Atlas (TCGA): an immeasurable source of knowledge. Współczesna Onkol.

[37] Battle A (2017). Genetic effects on gene expression across human tissues. Nature.

[38] Vivian J, Rao AA, Nothaft FA, Ketchum C, Armstrong J, Novak A, Pfeil J, Narkizian J, Deran AD, Musselman-Brown A (2017). Toil enables reproducible, open source, big biomedical data analyses. Nat. Biotechnol..

[39] Wang D, Qian X, Du Y-CN, Sanchez-Solana B, Chen K, Park B, Chen B, Jenkins L, Luo J, Tripathi BK (2022). Abstract 3912: cProSite: A web based interactive platform for on-line proteomics and phosphoproteomics data analysis. Cancer Res.

[40] Uhlén M, Fagerberg L, Hallström BM, Lindskog C, Oksvold P, Mardinoglu A, Sivertsson Å, Kampf C, Sjöstedt E, Asplund A (2015). 2015 Tissue-based map of the human proteome. Science.

[41] Dai G, Sun Y, Wei R, Xi L (2023). Small leucine-rich proteoglycan PODNL1 identified as a potential tumor matrix-mediated biomarker for prognosis and immunotherapy in a pan-cancer setting. Curr. Issues Mol. Biol..

[42] Dai M, Zhu X-L, Liu F, Xu Q-Y, Ge Q-L, Jiang S-H, Yang X-M, Li J, Wang Y-H, Wu Q-K (2017). Cholesterol synthetase DHCR24 induced by insulin aggravates cancer invasion and progesterone resistance in endometrial carcinoma. Sci. Rep..

[43] Wang Y, Gong H, Cao Y (2023). LncRNA WAC-AS1 expression in human tumors correlates with immune infiltration and affects prognosis. Hereditas.

[44] Cerami E, Gao J, Dogrusoz U, Gross BE, Sumer SO, Aksoy BA, Jacobsen A, Byrne CJ, Heuer ML, Larsson E (2012). The cBio cancer genomics portal: An open platform for exploring multidimensional cancer genomics data. Cancer Discov..

[45] Gao J, Aksoy BA, Dogrusoz U, Dresdner G, Gross B, Sumer SO, Sun Y, Jacobsen A, Sinha R, Larsson E (2013). Integrative analysis of complex cancer genomics and clinical profiles using the cBioPortal. Sci. Signal..

[46] Bonneville R, Krook MA, Kautto EA, Miya J, Wing MR, Chen H-Z, Reeser JW, Yu L, Roychowdhury S (2017). Landscape of microsatellite instability across 39 cancer types. JCO Precis Oncol..

[47] Chandrashekar DS, Bashel B, Balasubramanya SAH, Creighton CJ, Ponce-Rodriguez I, Chakravarthi BVSK, Varambally S (2017). UALCAN: A portal for facilitating tumor subgroup gene expression and survival analyses. Neoplasia.

[48] Li T, Fan J, Wang B, Traugh N, Chen Q, Liu JS, Li B, Liu XS (2017). TIMER: A web server for comprehensive analysis of tumor-infiltrating immunecells. Cancer Res..

[49] Zeng D, Ye Z, Shen R, Yu G, Wu J, Xiong Y, Zhou R, Qiu W, Huang N, Sun L (2021). IOBR: Multi-omics immuno-oncology biological research to decode tumor microenvironment and signatures. Front Immunol.

[50] Thorsson V, Gibbs DL, Brown SD, Wolf D, Bortone DS, Ou Yang T-H, Porta-Pardo E, Gao GF, Plaisier CL, Eddy JA (2018). The immune landscape of cancer. Immunity.

[51] Yoshihara K, Shahmoradgoli M, Martínez E, Vegesna R, Kim H, Torres-Garcia W, Treviño V, Shen H, Laird PW, Levine DA (2013). Inferring tumour purity and stromal and immune cell admixture from expression data. Nat. Commun..

[52] He F, Wang J, Liu L, Qin X, Wan Z, Li W, Ping Z (2021). Esophageal cancer: Trends in incidence and mortality in China from 2005 to 2015. Cancer Med..

[53] Machlowska J, Baj J, Sitarz M, Maciejewski R, Sitarz R (2020). Gastric cancer: Epidemiology, risk factors, classification, genomic characteristics and treatment strategies. Int. J. Mol. Sci..

[54] Lordick F, Janjigian YY (2016). Clinical impact of tumour biology in the management of gastroesophageal cancer. Nat. Rev. Clin. Oncol..

[55] Raufaste-Cazavieille V, Santiago R, Droit A (2022). Multi-omics analysis: Paving the path toward achieving precision medicine in cancer treatment and immuno-oncology. Front Mol. Biosci..

[56] Korenjak M, Zavadil J (2019). Experimental identification of cancer driver alterations in the era of pan-cancer genomics. Cancer Sci..

[57] Chen F, Wendl MC, Wyczalkowski MA, Bailey MH, Li Y, Ding L (2021). Moving pan-cancer studies from basic research toward the clinic. Nat. Cancer.

[58] Li S, Liu Y, Yao C, Xu A, Zeng X, Ge Y, Sheng X, Zhang H, Zhou X, Long Y (2023). Prognostic prediction and expression validation of NSD3 in pan-cancer analyses. Biocell.

[59] Li Z, Lv H, Zhang F, Zhu Z, Guo Q, Wang M, Huang C, Chen L, Zhang W, Li Y (2024). Systematic analysis of DNA polymerases as therapeutic targets in pan-cancers. Biocell.

[60] Fayad E, Eid RA, Abdulsahib WK, Zaki MSA, Binjawhar DN, Alshaya DS, Altalhi SA, Alsharif G, Elwishahy AH, El-shaer NH (2024). CKS2 and its interacting network induce tumor progression with adverse effects on patients’ survival in a pan-cancer model. J. Biol. Regul Homeost Agents.

[61] Stenmark H (2009). Rab GTPases as coordinators of vesicle traffic. Nat. Rev. Mol. Cell Biol..

[62] Xu T, Song X, Wang Y, Fu S, Han P (2020). Genome-wide analysis of the expression of circular RNA full-length transcripts and construction of the circRNA-miRNA-mRNA network in cervical cancer. Front Cell Dev. Biol..

[63] Obuchowski NA, Bullen JA (2018). Receiver operating characteristic (ROC) curves: review of methods with applications in diagnostic medicine. Phys. Med. Biol..

[64] Mandrekar JN (2010). Receiver operating characteristic curve in diagnostic test assessment. J. Thorac. Oncol..

[65] Han Y, Liu X, Ye H, Tian Y, Ji Z (2020). Lower mutant-allele tumor heterogeneity is a biomarker in FGFR3-mutant bladder cancer for better prognosis. World J. Surg. Oncol..

[66] Lawrence MS, Stojanov P, Polak P, Kryukov GV, Cibulskis K, Sivachenko A, Carter SL, Stewart C, Mermel CH, Roberts SA (2013). Mutational heterogeneity in cancer and the search for new cancer-associated genes. Nature.

[67] Galvano A, Gristina V, Malapelle U, Pisapia P, Pepe F, Barraco N, Castiglia M, Perez A, Rolfo C, Troncone G (2021). The prognostic impact of tumor mutational burden (TMB) in the first-line management of advanced non-oncogene addicted non-small-cell lung cancer (NSCLC): A systematic review and meta-analysis of randomized controlled trials. ESMO Open.

[68] Morgan AE, Davies TJ, Mc Auley MT (2018). The role of DNA methylation in ageing and cancer. Proc. Nutr. Soc..

[69] Bareche Y, Kelly D, Abbas-Aghababazadeh F, Nakano M, Esfahani PN, Tkachuk D, Mohammad H, Samstein R, Lee C-H, Morris LGT (2022). Leveraging big data of immune checkpoint blockade response identifies novel potential targets. Ann. Oncol..

[70] Wang C, Li Y, Jia L, Kim JK, Li J, Deng P, Zhang W, Krebsbach PH, Wang C-Y (2021). CD276 expression enables squamous cell carcinoma stem cells to evade immune surveillance. Cell Stem Cell.

